# White Matter Loss in a Mouse Model of Periventricular Leukomalacia Is Rescued by Trophic Factors

**DOI:** 10.3390/brainsci3041461

**Published:** 2013-11-12

**Authors:** Araceli Espinosa-Jeffrey, Socorro A. R. Barajas, Alfonso R. Arrazola, Alana Taniguchi, Paul M. Zhao, Payam Bokhoor, Sandra M. Holley, Don P. Dejarme, Brian Chu, Carlos Cepeda, Michael S. Levine, Pierre Gressens, Alfredo Feria-Velasco, Jean de Vellis

**Affiliations:** 1 Intellectual and Developmental Disabilities Research Center, Semel Institute for Neuroscience and Human Behavior, Department of Psychiatry, University of California Los Angeles, Los Angeles, CA 90095, USA; E-Mails: at.miyori@gmail.com (A.T.); zhaoyang17@yahoo.com (P.M.Z.); payambokhoor@ucla.edu (P.B.); sholley@mednet.ucla.edu (S.M.H.); dpdejarme@gmail.com (D.P.D.); aznhistrymaker@gmail.com (B.C.); ccepeda@mednet.ucla.edu (C.C.); mlevine@mednet.ucla.edu (M.S.L.); 2 University Center for Biological and Agricultural Sciences, University of Guadalajara, Guadalajara 44100, Mexico; E-Mails: barajasmercado@yahoo.com.mx (S.A.R.B.); aracafar@yahoo.com.mx (A.R.A.); alfredoferia1340@hotmail.com (A.F.-V.); 3 French Institute of Health and Medical Research (INSERM), U676, Robert Debré Hospital, Paris 75019, France; E-Mail: pierre.gressens@inserm.fr

**Keywords:** premature birth, excitotoxicity, periventricular leukomalacia, white matter regeneration and repair, central nervous system repair, transferrin, insulin and IGF-1.

## Abstract

Periventricular leukomalacia (PVL) is the most frequent cause of cerebral palsy and other intellectual disabilities, and currently there is no treatment. In PVL, glutamate excitotoxicity (GME) leads to abnormal oligodendrocytes (OLs), myelin deficiency, and ventriculomegaly. We have previously identified that the combination of transferrin and insulin growth factors (TSC1) promotes endogenous OL regeneration and remyelination in the postnatal and adult rodent brain. Here, we produced a periventricular white matter lesion with a single intracerebral injection of *N*-methyl-d-aspartate (NMDA). Comparing lesions produced by NMDA alone and those produced by NMDA + TSC1 we found that: NMDA affected survival and reduced migration of OL progenitors (OLPs). In contrast, mice injected with NMDA + TSC1 proliferated twice as much indicating that TSC1 supported regeneration of the OLP population after the insult. Olig2-mRNA expression showed 52% OLP survival in mice receiving a NMDA injection and increased to 78% when TSC1 + NMDA were injected simultaneously and ventricular size was reduced by TSC1. Furthermore, in striatal slices TSC1 reduced the inward currents induced by NMDA in medium-sized spiny neurons, demonstrating neuroprotection. Thus, white matter loss after excitotoxicity can be partially rescued as TSC1 conferred neuroprotection to preexisting OLP and regeneration via OLP proliferation. Furthermore, we showed that early TSC1 administration maximizes neuroprotection.

## 1. Introduction

Myelin is the essential fatty membrane that provides insulation to nerve fibers in the brain and spinal cord. In addition, myelin ensures the normal and adequate transmission of electrical signals from the brain or spinal cord to specific parts of the body and *vice versa*. Motor, sensory and integrative functions depend on the rapid, seamless conduction of nerve impulses along these myelinated nerve fibers [1]. In the developing brain, oligodendrocytes (OLs) are cells that form and maintain myelin throughout life. The cells are generated during perinatal life, and immature OLs are particularly vulnerable to insults, such as lack of oxygen. The impairment of OL development leads to intellectual and lifelong disabilities. Currently, treatments for myelin disorders are limited to reducing the inflammatory responses induced by hypoxia or glutamate toxicity and alleviating the symptoms, but little repair and gain-of-function are achieved. In the developing brain, OL maturation occurs perinatally, and immature OLs are particularly vulnerable.

Myelin loss can result from myelin damage, such as that observed in multiple sclerosis (MS) or after brain or spinal cord injury. The lack of myelin could also be inherited, such as in Pelizaeus-Merzbacher, a leukodystrophy that reflects all known types of mutations and gene duplication in the proteolipid gene (PLP). Both types of myelin deficiencies are progressive and lead to dysfunction, paralysis and often death. Due to improved neonatal intensive care, the number of low birth weight (<1500 g) infants that survive is increasing [2,3]. Approximately 13 million premature infants are born worldwide every year, of which 90% survive beyond infancy. Premature infants, however, are extremely vulnerable to brain injury, and ~5%–10% of the survivors develop cerebral palsy and 40%–50% develop cognitive and behavioral deficits. The pattern of perinatal brain injury is age-dependent, e.g., while in full-term infants the cerebral cortex is predominantly affected through neuronal loss, in premature infants cerebral white matter injury (WMI) is more frequent. [4]. An array of brain injuries is included under the umbrella of cerebral WMI, of which periventricular leukomalacia (PVL) is one of the most severe forms [5]. Neuroimaging studies indicate that the most predominant type of PVL is diffuse WMI, resulting from the disrupted maturation of OL progenitors (OLPs), leading to hypomyelination [6,7]. Perinatal WMI is a leading cause of cognitive and functional disability and is detected in a significant percentage of preterm human infants. Prematurity is often the most important risk factor for brain injury because the white matter is formed in the perinatal period, thus becoming the primary target of hypoxia-ischemia lesions resulting in PVL and germinal matrix/intraventricular hemorrhage, associated with a loss of gray matter [2,3,4,8].

Injury to both gray and white matter is the primary focus for improving neurological outcomes in survivors [9]. Several factors contribute to WMI and PVL, among which is an immature vascular system in the deep periventricular regions of the brain, making this organ vulnerable to cerebral ischemia. It is thought that in preterm infants, blood flow is low; consequently, any decrease in peripheral blood pressure exposes the brain to hypoxic-ischemic episodes, nonetheless, the evidence supporting this belief is scarce. Hypoxic-ischemic events trigger free radical formation and increase extracellular glutamate released from injured axonal processes, with a concomitant decreased uptake by astrocytes. OLs express functional glutamatergic receptors of the *N*-methyl-d-aspartate (NMDA)-subtype, and OLPs and pre-OLs are sensitive to both oxidative stress and glutamate-induced excitotoxicity [10,11,12,13]. Moreover, NMDA receptors are involved in OL death following ischemia [14,15]. Animal models mimicking PVL have been developed, facilitating the identification of the primary pathways that lead to OL cell death and WMI [12,13].

Previously, using a proteolipid protein rodent mutant, the myelin-deficient (*md*) rat, we showed that the failure of OLs to mature reflects myelin deficiencies [16]. We also showed that in cell culture, *md* OLPs express markers synthesized by more mature OLs. However, these markers were not expressed *in vivo* [17], suggesting that an adequate trophic environment may promote further maturation of OLs in these mutants. We then compared the ontogenetic profile for *transferrin* (*Tf*) gene expression in *md* mutants with that of unaffected rat pups through Northern blot analysis and *in situ* hybridization. Surprisingly, the synthesis of this early marker of OLs [18,19] was not observed in mutant OLs, while synthesis was normal in the choroid plexus, the second source of Tf in the central nervous system (CNS). Next, we demonstrated that a single apo-transferrin intraparenchymal injection administered to P5 rat pups enabled mutant OL to synthesize myelin basic protein (MBP) and myelin, indicating that Tf affects mutant OL maturation, regardless of its source; thus, Tf is an essential factor for myelination [20]. However, the *md* rat has a severe hypomyelinated CNS, and therefore, we sought to determine whether treatment with Tf in combination with other factors, would increase myelination. Using run-off transcription, we showed that Tf up-regulates the transcription of the *MBP* gene [21] and that it synergizes with IGF-1 increasing myelination. We named the combination of IGF-1 and Tf “TSC1”. This mixture promotes the maturation of endogenous OLs, facilitating the myelination of axons in the myelin deficient rat. Considering the adverse events leading to lifelong disabilities in the brain of premature neonates, in the present study, we investigated the potential preclinical therapeutic use of TSC1 to reduce excitotoxic injury and preserve the integrity of motor pathways in a model of PVL induced by intracerebral NMDA injection.

## 2. Experimental Section

### 2.1. Animals and Intraparenchymal Injection Procedures

Nestin-GFP mice [22] were bred at the University of California Los Angeles (UCLA, Los Angeles, CA, USA) in a restricted access, temperature-controlled vivarium under a 12 h:12 h light:dark cycle, with access to food and water *ad libitum*. Postnatal day four (P4) nestin-GFP mice pups were used. Each experiment consisted of three time points and five conditions: Non-treated mice, Saline, NMDA, NMDA + TSC1 simultaneously injected (N + TSC1 0 day) and NMDA + injection of TSC1 delayed 3 days (N + TSC1 3 day). The stereotaxic intraparenchymal injections in brain, collection and sectioning of tissues for the characterization of cell phenotypes were performed as described [23]. Assessment of cellular stress was monitored through HSP-32 expression. We used 6 mice per condition. Three separate experiments were performed to assess the effects of NMDA alone or with TSC1 on endogenous OLP survival, proliferation and maturation. Intraparenchymal injections were performed according to previously described methods [23,24].

Briefly, nestin-GFP P4 pups were injected as follows. Ophthalmology microsurgery instruments and a stereotaxic apparatus with an adjustable adapter for small (newborn) animals were used. The animals were anesthetized with halothane and placed on a stereotaxic frame for the intraparenchymal administration of the cells with a Hamilton syringe. The injection coordinates were 1.2 mm lateral (right) and 0.7 mm caudal to Bregma. The needle was inserted 2.5 mm deep into the corpus callosum (CC). The control group consisted of nestin-expressing mice that received saline injections, using the same procedures. We performed unilateral grafts. The total volume of the injected factors was 1.5 μL.

### 2.2. Induction of Glutamate Excitotoxicity (GME)

We induced excitotoxicity in the GFP-nestin mice through the stereotaxic injection of NMDA into the CC. A single unilateral injection was performed on P4 mice as previously described [23].

### 2.3. Preparation of Trophic Factors

The stock solutions were prepared in saline, and the TSC1 cocktail was also prepared in saline.

### 2.4. Collection and Examination of the Samples

Brain collection and sectioning for the characterization of cell phenotypes was performed as previously described [23]. To determine the effectiveness of the treatment, OLs and myelin were determined through the expression of cyclic nucleotide 3′-phosphohydrolase (CNPase). Cell proliferation was determined using Ki67, a marker of cellular proliferation, in combination with cell and stage specific markers for OLs.

### 2.5. Measurements of the Lateral Ventricles

Para-coronal 20 μm frozen sections were rinsed and mounted for lateral ventricle measurements. The area of ipsilateral “IL” *vs.* contralateral “CL” ventricles was obtained using LSM-Meta software (Zeiss, Jena, Germany), and the difference was calculated in μm^2^. The data were converted to a percentage of the area, with respect to the contralateral ventricle.

### 2.6. *In Situ* Hybridization

Non-radioactive *in situ* hybridization for Olig2 was performed as previously described [23,25]. The cells were counted using the interactive AxioVision Rel 4.6 program from Zeiss.

The immunofluorescence procedures were performed as previously described [23,26]. Briefly, cultures were fixed with 4% paraformaldehyde. Samples were blocked for 1 h in 1% BSA (Sigma-Aldrich, Buchs, Switzerland), 0.3% Triton X-100 (Sigma-Aldrich), and 10% normal goat or donkey serum in PBS. Primary antibodies were diluted in carrier solution (1% BSA and 0.3% Triton X-100 in PBS) and incubated overnight at 4 °C. After washing with PBS, secondary antibodies were incubated for 1 h at room temperature, washed with TBS and mounted. Samples were imaged using the LSM 510 META confocal microscope (Zeiss) and analyzed with the Axiovision software (Zeiss).

### 2.7. Myelin Staining for Frozen Sections: Spielmeyer’s Method

Staining was performed using hematoxylin for 10–24 h, followed by rinsing in distilled water. The tissues were subsequently differentiated in 2.5% ferric ammonium sulfate solution, washed thoroughly for 2 h, followed by dehydration and mounting [27].

### 2.8. Stereology

For stereology, brain sections were 28 μm thick. We used an Axio-Imager M2 (Zeiss) microscope equipped with the Stereo-Investigator software [Micro Bright Field (MBF) Inc., Williston, VT, USA]. The entire ventricular region (of the ipsilateral ventricle) was selected and imaged using a 5× objective and the Cavalieri Probe (MBF).

### 2.9. Examination of the Neuroprotective Effects of TSC1 against NMDA Excitotoxicity

For these experiments, brain slices were incubated in artificial cerebrospinal fluid (ACSF) or in ACSF supplemented with TSC1 prior to the acute exposure to NMDA. Striatal medium-sized spiny neurons (MSNs) were recorded using the patch-clamp technique, and the responses were evoked through electrical stimulation or bath application of NMDA (50–100 μM). Responses were compared in control slices (incubated in vehicle) and in slices incubated for 1 h in TSC1 solution. The amplitude of the response was evaluated using pClamp software (Molecular Devices, Sunnyvale, CA, USA). Coronal slices of 300 μm thickness were used. Whole-cell patch clamp recordings were obtained from neurons in the dorsal striatum. The cells were visualized through infrared-differential interference contrast (IR-DIC) microscopy and identified according to somatic size and basic membrane properties. The patch pipette (4–6 MΩ) contained a Cs-methanesulfonate (CsMeth) internal solution composed of: CsMeth 125 mM, NaCl 4 mM, KCl 3 mM, MgCl_2_ 1 mM, EGTA 9 mM, HEPES 8 mM, MgATP 5 mM, TrisGTP 1 mM, disodium phosphocreatine 10 mM and leupeptin 0.1 mM (pH 7.2; 270–280 mOsm/L) for recording in voltage clamp mode. The basic membrane properties were determined in cells perfused in standard ACSF at room temperature using the membrane properties function within Clampex 8.2 (Molecular Devices). To evoke synaptic currents, a monopolar glass stimulating electrode (patch-pipette filled with ACSF, impedance ~1 MΩ) was placed in the deep cortical layers or white matter approximately 200–300 μm from the recorded cell. The cell membranes were voltage clamped at +40 mV to measure NMDA synaptic responses. When NMDA was applied in the bath, cell membranes were held at −70 mV.

### 2.10. Statistical Analysis

Quantitative data are expressed as the means ± SE. Comparison of mean values between multiple groups was evaluated using ANOVA followed by a *post hoc* Tukey HSD multiple comparison test where data obtained with the various treatments were compared either to their respective saline control or non-treated samples within the same age group. Significance was assumed when *p* ≤ 0.05. Student’s *t* test was used to analyze electrophysiological data.

## 3. Results

### 3.1. Effect of TSC1 on Ventriculomegaly

**Figure 1:**
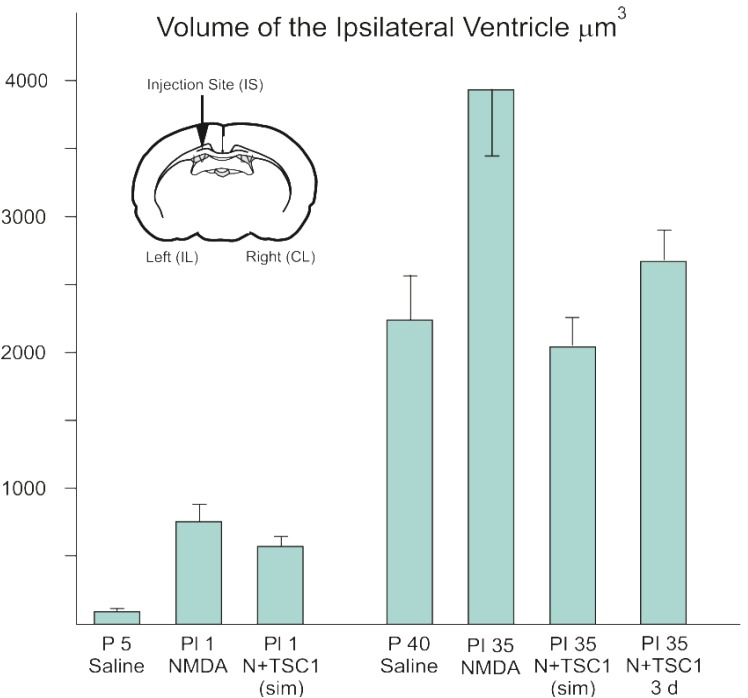
The enlargement of the ventricle after excitotoxicity is decreased with a single dose of TSC1. Seven and 35 days after injection of the specific treatments para-coronal, 28 μm frozen sections were rinsed and mounted for stereological measurements. The volume of the entire ipsilateral (IL) ventricular region was selected and imaged using a 5× objective and the Cavalieri Probe (MBF) obtained in μm^3^. Data represent measurements of 6 adjacent coronal sections per time point from three separate experiments. The diagram shows a coronal section and the arrow points at the injection site (IS). Values are expressed as mean ± SEM of three independent experiments. *p* ≤ 0.05 *vs.* controls. All differences across treatments were significant. Abbreviations: P5 = Postnatal day five; PI = post injection day.

The wall of the ventricles and subventricular region is the source of uncommitted neural progenitors both in the perinatal and the adult brain. Therefore, we determined the impact of NMDA in this region. We examined para-coronal sections to measure the ventricular size of GFP-nestin transgenic mice either untreated or injected with saline, NMDA alone, NMDA + TSC1 injected simultaneously, or NMDA followed by a delayed (3 days) injection of TSC1. Frozen sections (28 μm) were cut, washed, and mounted to be examined under the AxioVision microscope (Zeiss) equipped with the Stereo Investigator software (MBF). The volume of the ipsilateral ventricle was obtained using the Cavalieri Probe (MB field) (data are shown in Figure 1). The values from six adjacent coronal sections per time point, per mouse were obtained from three separate experiments. The mean values of these six sections were considered the value per mouse, per treatment for its respective experiment. Seven days after intraparenchymal injections, the animals treated with NMDA alone showed 82% larger ipsilateral ventricle (ILV; injected hemisphere) with respect to the contralateral ventricle (non-injected hemisphere). When NMDA was injected with TSC1 simultaneously (N + TSC1 sim), a reduction in the ventricle enlargement was observed; the ILV was 64% larger than the CLV (Figure 1). Upon insult, an acute inflammatory response that interferes with the beneficial effects of TSC1 is typically observed. Thus, we also examined whether a delayed injection of TSC1 would increase the neuroprotective effect if injected after cell death and the active inflammatory events occur within hours after the insult. We observed that the enlargement of the ILV was reduced but to a lesser extent in mice receiving a 3-day delayed TSC1 injection when compared to mice that got the combined treatment simultaneously (Figure 1).

### 3.2. Effects of TSC1 on OL (Oligodendrocyte) Proliferation

**Figure 2:**
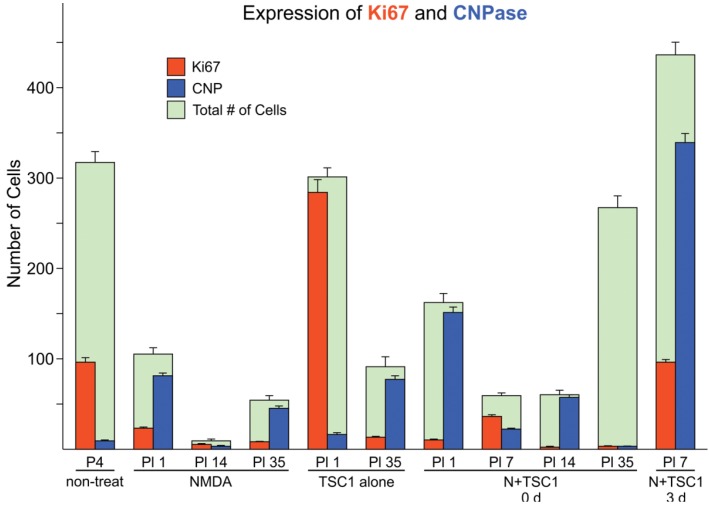
Cell loss in the subventricular zone (SVZ) is partially rescued in the presence of TSC1 via cell survival or cell proliferation. Double immunofluorescence: cells expressing the proliferation marker Ki67 were found in this region at early time points in the presence of TSC1. Some cells co-expressed the two markers (Ki67/CNPase). In contrast, when *N*-methyl-d-aspartate (NMDA) was injected alone there was a dramatic reduction of the total number of cells. Green bars represent the total number of cells (*i.e.*, 100% or the total number of cells counted in that field. Numbers for saline and non-treated mice were very close with no significant differences. Values are expressed as mean ± SEM of the counts of 9 fields per area from three independent experiments. *p* < 0.05 *vs.* controls. All differences with respect to non-treated mouse brains as well as, across treatments were significant. P5 = Postnatal day five; PI = post injection day.

We used the marker Ki67 to assess cell proliferation. One day after NMDA treatment (PI-1), the cell loss was considerable after NMDA injection, showing on average 70% of cells expressing CNPase in the subventricular zone (SVZ). At this time point, in the SVZ non-treated mice displayed some cells expressing CNPase as cells may have already migrated into the parenchyma, and 30% of the total number of cells were Ki67-positive. Mice treated with NMDA alone showed basically an almost total demise of cells 24 h after the injection in the SVZ, showing only 30% of the total cell number observed in non-treated mice. Among the surviving cells, most were CNPase-positive and only a small fraction was positive for Ki67 (Figure 2). The subsequent time points, post-injection days 14 and 35, showed a reduction in both Ki67 and CNPase and the total number of cells in the SVZ. The total number of cells in mice injected with TSC1 alone at 24 h after the injection was equivalent to that in non-treated mice. Nonetheless, most of these cells proliferated, as shown by the location of Ki67, and few cells were CNPase-positive. Thirty-five days after TSC1 injection, most cells in the SVZ were CNPase-positive and only a fraction of these cells expressed Ki67. When both NMDA and TSC1 were injected simultaneously, the survival of CNPase-expressing cells present at the time of the excitotoxic insult was evident, while Ki67 labeling was reduced compared with non-treated mice at an equivalent age. Seven days after treatment, almost no CNPase-expressing cells were observed in the SVZ, from which most cells were progenies, showing co-expression of Ki67/CNPase. At 1 and 7 days after treatment (PI 1 and PI 7), most CNPase-expressing cells were Ki67-negative, suggesting that these cells were present prior to administration of N + TSC1 and these cells were the predominant population in the SVZ at these time points. At PI 35, many cells were present in the SVZ but not labeled with either marker. Most CNPase- and Ki67-expressing cells were absent from this region, suggesting that most OLs had migrated into the parenchyma by this time point. Interestingly, when NMDA was injected at P4, followed by an injection of TSC1 3 days later, the total number of cells, those CNPase-positive cells and progenies labeled with Ki67, was higher with respect to that found in mice injected with NMDA + TSC1 simultaneously, as shown in Figure 2.

### 3.3. Olig2 Expression

**Figure 3:**
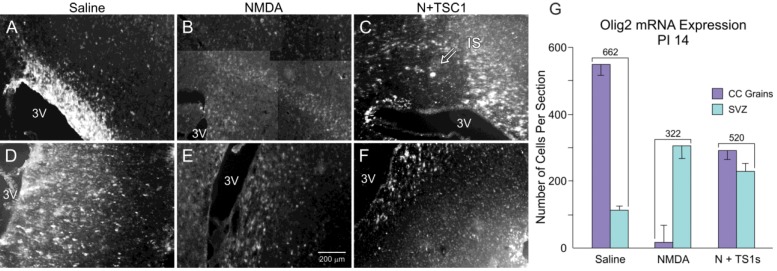
Panel (**A**–**F**). More Olig 2 expressing cells survive after NMDA exposure in the presence of TSC1. Examples of expression of the transcription factor Olig2 in the corpus callosum (CC) and SVZ 14 days after injection. Animals injected with saline displayed a large number of Olig2-positive cells in the CC (**A**) and a reduced number in the sub-ventricular zone (SVZ, **D**) suggesting that the vast majority of OLP had already migrated to the CC. (**B**) The CC of NMDA injected mice showed fewer cells and less intensely labeled cells while some intensely labeled cells were found in the SVZ (**E**). (**C**) the CC of the mouse injected with NMDA + TSC1 showed an extensive area containing Olig2-mRNA, this particular section shows the injection site (IS) devoid of Olig2-expressing cells but surrounded by intensely labeled OL progenitors. (**F**) Some Olig2-expressing cells were still present in the SVZ. (**C**) View of the injection site at the level of the CC of a mouse co-injected with NMDA and TSC1. The intensity of the label was low and the number of cells appeared reduced in both regions when compared to saline injected mice. (**G**) Quantitative data of Olig2-expressing cells. In animals injected with NMDA alone, the total number of Olig2-expressing cells in the CC and SVZ was reduced when compared to the total number of cells present in mice injected with saline. In contrast, there were more Olig2-expressing cells in the SVZ, than in animals injected with saline. Interestingly, with the N + TSC1 injection the total number of Olig2-positive cells in the CC and the SVZ was partially restored to 78% and almost to an equal distribution in the SVZ and the CC, suggesting that Olig2 positive cell migration was not disrupted by NMDA in the presence of TSC1. Values are expressed as mean ± SEM of the counts of 6 fields per area from three independent experiments. *p* ≤ 0.05. All differences across treatments were significant.

We subsequently examined the expression of the transcription factor Olig2 through *in situ* hybridization as an indicator of the survival and migration of OLPs across treatments. The examination of sagittal sections at 14 days after the injection of the various agents showed that saline-injected mice displayed a large number of Olig2-positive cells in the CC (Figure 3A) and a reduced number in the SVZ, indicating that the vast majority of OLPs had previously migrated to the CC. In animals injected with NMDA alone, the number of Olig2-expressing cells in the CC was reduced to approximately 6% (20 cells) of the total number of cells present in mice injected with saline (562 cells). Nonetheless, there were more Olig2-expressing cells in the SVZ than in animals injected with saline. Interestingly, after the NMDA + TSC1 injection, the cumulative number of Olig2-expressing cells, in both the CC and the SVZ, was greater than that of mice injected with NMDA alone (520 cells *vs.* 322 cells), suggesting that the migration of Olig2-expressing cells was not disrupted with NMDA treatment in the presence of TSC1 (Figure 3G).

### 3.4. Myelination after Treatment

**Figure 4:**
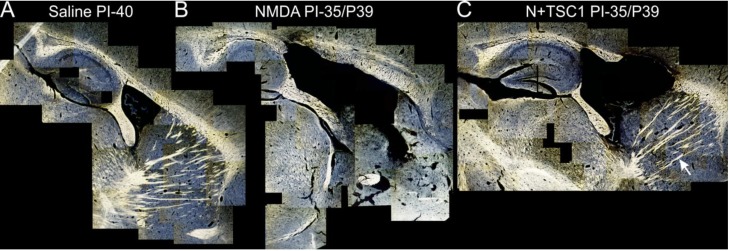
Myelination is partially rescued from excitotoxicity by TSC1. Representative para-sagittal brain sections (28 μm thick) stained with the Spielmeyer’s method for frozen sections. These views show (**A**) the myelination pattern with saline treatment 40 days post-injection (PI). The extent of tissue damage, ventricle enlargement, and myelin loss in mice treated with NMDA (**B**) and its recovery with TSC1 (**C**) treatment. Moreover, NMDA treated mice showed areas where tissue was spared in the CC and CPu but there was not myelin staining. In contrast, mice treated with TSC1 showed nice myelinated fibers indicating that after the excitotoxic insult functional OLs developed and actively myelinated axons. The arrow points to a bundle of myelinated axons in the CPu of a mouse treated with NMDA + TSC1 simultaneously. In contrast, the same region of mice injected with NMDA alone did not show myelinated axons. The variability within each treatment group, consisting of 6 animals, was minimal based on low magnification examination as shown in Figure 4.

The aim of this study was to determine at a qualitative level whether myelination could be restored after an excitotoxic insult using TSC1. Thirty-five days after treatment, we examined parasagittal brain sections obtained from mice receiving the various treatments. The Spielmeyer’s myelin staining method was used. We observed that, similar to the ILV of mice injected with NMDA alone or with NMDA + TSC1, the third ventricle was also enlarged. However, the proportion of enlargement was reduced in the presence of TSC1, whether injected simultaneously or after a 3-day delay. In mice treated with NMDA alone some regions where tissue was spared appeared devoid of myelin. In contrast, mice treated with NMDA + TSC1 showed more myelinated fibers in the CC and the striatum demonstrating the extensive neuroprotective effect of TSC1 (Figure 4).

### 3.5. Expression of the Stress Protein HSP-90 in Acute Brain Slices

**Figure 5:**
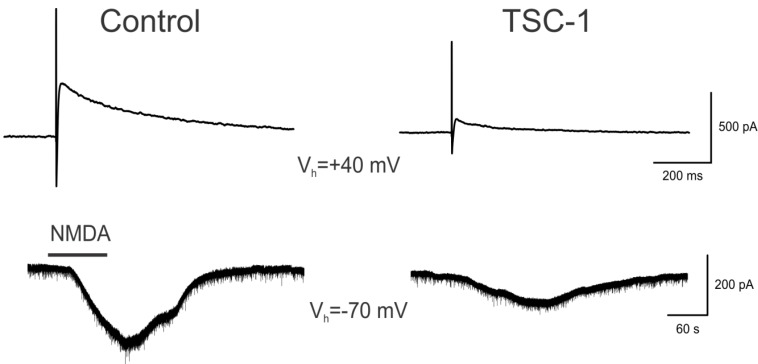
Neuroprotection of Medium-sized Spiny Neurons (MSNs). Upper traces represent responses of striatal MSNs evoked by electrical stimulation (0.2 mA, 0.1 ms duration) of cortical inputs. Recordings were obtained with patch electrodes in voltage clamp mode (holding voltage at +40 mV). Control trace is a NMDA receptor-mediated response recorded in normal ACSF solution and isolated pharmacologically by adding 2,3-dihydroxy-6-nitro-7-sulfamoyl-benzo[*f*]quinoxaline-2,3-dione (NBQX, 10 μM) and Bicuculline (10 μM). The trace on the right is from another cell recorded after the slice was incubated for 1 h in TSC1 solution (2 μL/mL). Lower traces show responses to bath application of NMDA (100 μM) in ACSF (left) or after incubation for 1 h in TSC-1. Notice that the NMDA responses were significantly reduced compared to control conditions. Calibration bars apply to both traces.

**Figure 6:**
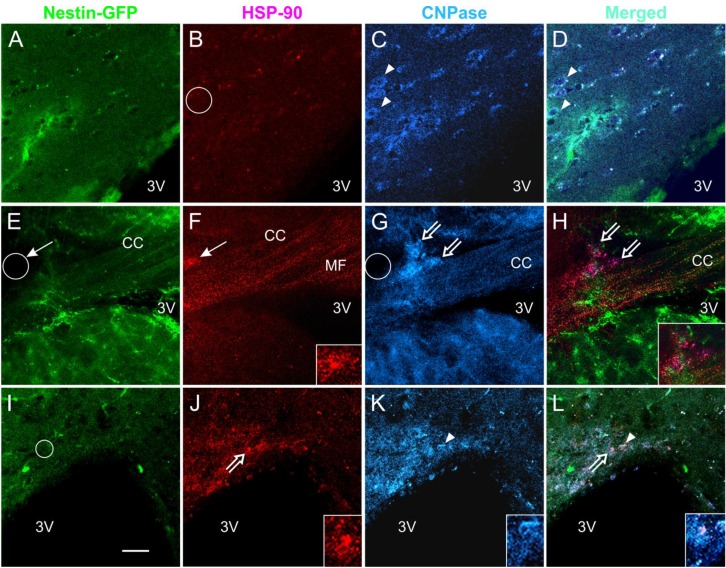
Acute NMDA exposure elicits the expression of HSP-90 in the CC and TSC1 neutralizes the NMDA effect. Coronal brain slices 300 μm thick were used for the acute treatment of NMDA alone or in slices pre-incubated with TSC1. After electrophysiology, slices were fixed and immunolabeled for the cell stress marker HSP-90 and the OL marker CNPase. (**A**–**D**) representative views of untreated slices, neither nestin (**A**) nor CNPase-expressing cells (**C**-arrowheads) were labeled for HSP-90 (**B**-circle). (**D**) merged image. After acute NMDA, CNPase-positive OL expressed HSP-90 (**E**–**F**). Moreover, cells that were not labeled for either of the two markers also expressed HSP-90 (**F**,**G**-thin arrows and circle). In slices pre-incubated with TSC1 followed by acute NMDA treatment (see methods for details) (**I**–**L**) the majority of CNPase-positive cells did not express HSP-90 (**K**). Nestin-positive cells did not express HSP-90 in the presence of TSC1 (**I**-circle). Some cells co-expressed CNPase and HSP-90 (**J**,**K** and **L**-open arrows). Insets show higher magnification views of the cells pointed by open arrows in (**J)** and (**L**). Arrowhead points to a CNPase-positive cell (**K**) that co-expresses HSP-90 (**L**). The inset in (**J**) shows an example on the colocalization of both HSP90 and CNPase both, in the cell body and processes (**K**,**L**). Calibration bar in **(I)** corresponds to 50 μm.

Coronal slices were exposed to either NMDA alone or pre-incubated for 1 h to 2 h in TSC1 and subsequently exposed to NMDA. After electrophysiology as shown (Figure 5), slices were fixed and examined through double immunofluorescence for the cell stress marker heat shock protein 90 (HSP-90) and the OL marker cyclic nucleotide 3′-phosphohydrolase (CNPase). The results showed that HSP-90 was expressed in the CC white matter in NMDA-treated mice but not in non-treated slices (Figure 6A–D *vs.* Figure 6E–L respectively). Those slices that were pre-incubated with TSC1 presented a clear expression of HSP-90 at the level of the cell soma and processes but not along axonal fibers. CNPase expression was observed and not all CNPase positive cells expressed HSP-90. In contrast, slices treated with NMDA without pre-incubation in TSC1 showed a diffuse distribution of HSP-90 preferentially along the fibers expressed in mini-compartments like “puncta” and only few inter-fascicular cells were clearly seen expressing this stress protein but not CNPase (Figure 6F,G).

### 3.6. Coronal Views of the Caudate Putamen in Nestin-GFP Mouse Slices: Acute Treatment

**Figure 7:**
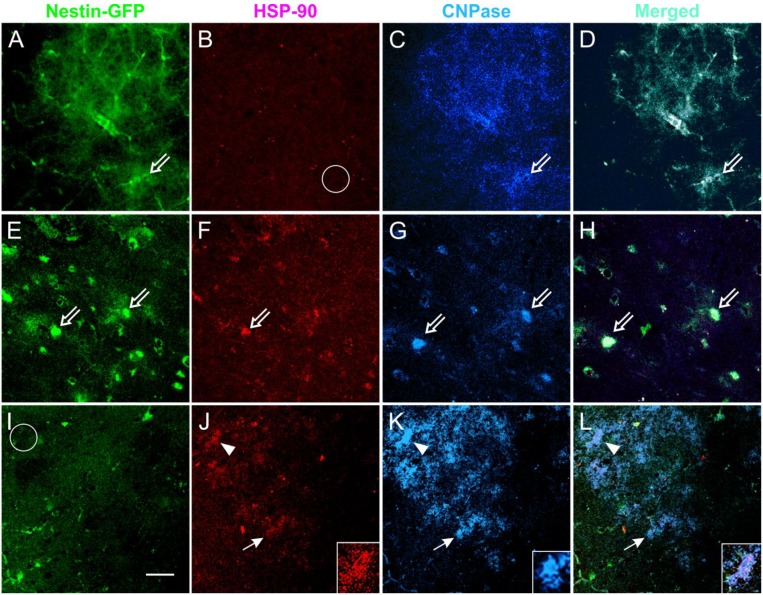
Acute NMDA elicits the expression of HSP-90 in the CPu and TSC1 neutralizes the NMDA effect. Views of the CPu of 300 μm coronal slices used for the acute treatment of NMDA alone or in slices pre-incubated with TSC1. After electrophysiology, slices were fixed and immunolabeled for the cell stress marker HSP-90 and the OL marker CNPase. HSP-90 was expressed in some cells located in the CPu white matter. Non-treated slices (**A**–**D**) displayed nestin-expressing cells that were negative for HSP-90 (**B**–**empty circle**). Slices directly exposed to NMDA (**E**–**H**); showed colocalization of nestin and HSP-90 (**E**,**F**) and few cells co-express the three markers (**open arrows**). CNP-expressing cells lost processes or expression of CNPase in their processes. Slices incubated with TSC1 prior to NMDA. TSC1 appeared to maintain the tissue in a mild stage of stress with fewer nestin-expressing cells co-expressing HSP-90 (panel **I**) and increased number of CNPase-positive OL (**K**). Arrowheads show cells co-expressing HSP-90 and CNPase (**K**). Insets show detail of the label distribution of the cell marked with arrowheads, the merged view allows for the visualization of HSP-90 in the cell soma while CNPase is distributed in the cell body and numerous cell processes (**L**). **K**-inset shows a healthy OL that did not express HSP-90 (**I**-**circle**).

After electrophysiology, coronal brain slices (300 μm thickness) were fixed and examined by double immunofluorescence for the cell stress marker HSP-90 and cyclic nucleotide 3′-phosphohydrolase (CNPase), an oligodendrocyte marker. The double staining showed that HSP-90 was not expressed in non-treated slices. In contrast, HSP-90 was expressed as “puncta” mainly along myelin tracks. Some OLs recognized by CNPase co-expressed HSP-90 in NMDA treated slices. Slices pre-incubated in TSC1 had HSP-90 expressing cells that co-expressed CNPase, but the myelin tracks were not labeled by the stress protein, suggesting neuroprotection through TSC1 (Figure 7).

### 3.7. TSC1 Decreases NMDA-Induced Currents

To examine the mechanisms through which TSC1 rescued some of the impairments produced by NMDA injections, we performed electrophysiological experiments using brain slices. Bath application of NMDA (50–100 μM) induced inward currents in all striatal MSNs examined (*n* = 16, 8 controls and 8 TSC1-treated). Most cells (*n* = 6) recorded after 1–2 h incubation in TSC1 showed a reduction in NMDA currents compared with the controls, 1 cell showed no change and 1 cell treated with TSC1 showed a slight increase (20%). The average percent reduction was 63% (*t* = 2.55, *p* < 0.02). As bath application affects both synaptic and extrasynaptic NMDA receptors, we also tested the effects of TSC1 incubation in a pair of MSNs. The cells were held at +40 mV and the responses were evoked by electrical stimulation of the CC. The cell incubated in TSC1 showed much smaller NMDA currents, confirming the neuroprotective effects of TSC1 (Figure 5).

## 4. Discussion

Injury or developmental damage to the white matter during formation in the immature brain leads to substantial motor, cognitive, and learning deficits. Premature birth is a major cause, and the initial insult may be followed by secondary and tertiary mechanisms, resulting in further deterioration of the brain structure and function [28]. It has been suggested [28] that these tertiary mechanisms of damage include persistent inflammation and epigenetic changes that can be modulated from being deleterious to becoming an opportunity for CNS repair. This reasoning has prompted research aimed at designing new therapies to treat individuals suffering from PVL and cerebral palsy. The goal of the present study was to evaluate endogenous repair of the immature brain in a mouse model of PVL. Based on the results from our previous work on myelin repair in dysmyelinating and demyelinated adult mice [23,26,29], we explored the therapeutic potential of TSC1 using a mouse model of white matter injury, as most commonly observed in premature infants. NMDA receptors are expressed in developing OL cell processes, and these receptors mediate excitotoxic injury [16]. We evaluated different parameters: ventriculomegaly, cell proliferation, expression of OL markers Olig2 and CNPase, the extent of myelination, and excitotoxic responses.

Ventriculomegaly occurs in the premature newborn [30]. Following transient excitotoxicity in the neonatal brain, a series of events evolve temporally and spatially, leading to long-lasting disabilities. Without diminishing the importance of cell replacement therapies via transplantation, our recent efforts have been focused on the activation of endogenous neural regeneration to foster endogenous repair mechanisms via activation of progenitor cells to promote myelination. Cerebral palsy is a major health problem caused by brain damage during pregnancy, delivery, or the immediate postnatal period. Perinatal stroke, intraventricular hemorrhage, and asphyxia are the most common causes of neonatal brain damage. PVL is predominantly observed in premature infants and is the most common predecessor of cerebral palsy [28]. OLP treatment *via* transplantation has proven effective in restoring injured organs and tissues in animal models. Our data showed that TSC1, injected simultaneously with NMDA or three days after the excitotoxic insult, considerably reduced the extent of ventricular enlargement, with a concomitant increase in CNPase-expressing cells.

### 4.1. Proliferation of OLs after Treatment

We used the proliferation marker Ki67 as an analog of BrdU in combination with the enzyme CNPase. We selected CNPase to identify OLs because it allows for the visualization of OL cell bodies. CNPase accounts for ∼2%–5% of the total protein in the CNS myelin and 0.5%–1% of peripheral nervous system myelin [31]. CNP catalyzes the hydrolysis of 2′,3′-cyclic nucleotides to form the corresponding 2′-monophosphates. CNP has been identified as an integral protein of myelin, produced by OLs in the CNS [31,32,33]. Although this enzyme has also been observed in non-myelin membranes derived from various organs, including the liver, thymus, adrenal glands, kidney, heart, and skeletal muscle, the levels of the enzymatic activity are significantly lower than those in the CNS [33,34,35]. Therefore, CNPase is an excellent marker for myelinogenesis in both the CNS and PNS. In the present work, this marker facilitated the identification of cells that evolved from the Olig2 stage to a more mature stage, and this transition allowed us to observe the direct effect of TSC1 as a neuroprotector of the Olig2-existing population at the time of the excitotoxic insult, as these cells appeared to move forward in the OL lineage.

### 4.2. TSC1 as Regulator of Myelination

OL development is critically dependent on the presence of extracellular trophic factors. Several key factors have been identified through the study of myelin mutant animals and cell culture studies [36]. We previously showed that normal OLs synthesize and secrete Tf, whose levels are significantly reduced in the CNS of myelin mutant animals, such as the md rat, a PLP mutant [19] and the Long Evans shaker (LES) rat, a *MBP* mutant [37,38]. Because of the inadequate expression levels of *Tf* in OLs, while normal levels are maintained in the epithelial cells of the choroid plexus, OL maturation and myelination are inefficiently supported [37]. In addition, we have demonstrated that, by restoring Tf levels in the md brain at P5, endogenous OLs were mature and formed thin myelin sheaths within 14 days [20]. Similar studies using wild-type animals have shown that increasing Tf levels through intrathecal injection results in premature OL maturation and myelination [39,40]. This result was also confirmed through Tf over-expression in the brain of transgenic mice [41], demonstrating the important role that Tf plays on myelinogenesis and maintenance. Another factor impacting OL development is insulin growth factor-1 (IGF-1). IGF-1 is a pleiotropic factor with diverse roles in the CNS [42]. IGF-1 is a potent promoter of proliferation of neural precursor cells and promotes subsequent steps in the differentiation of neurons, astrocytes and oligodendroctyes. In addition to proliferation, IGF-1 has been shown to promote myelination [43,44]. IGF-1 is widely distributed in the fetal and neonatal CNS, but it is restricted in the adult CNS [45]. Similarly, IGF-1 receptor expression is heterogeneous during early stages of CNS development, but the expression levels decline postnatally [46] reaching low levels in the aging brain. Furthermore, a partial deletion of the *IGF-1* gene results in intrauterine growth failure and severe post-natal growth delays and intellectual disabilities [47]. Based on these facts, the present work was performed to test the potential of TSC1 to ameliorate white matter injury after excitotoxicity, as one of many components of PVL. The results showed a beneficial effect on the survival of pre-existing CNPase-positive OLs and the proliferation of these cells in the presence of a single injection of TSC1. Similar to studies performed with the *md* mutant rat, TSC1 triggered both cell proliferation in the SVZ and the migration of Olig2-positive cells into the CC and CPu, where these cells are expected to myelinate naked axons. These results confirm that TSC1 is also beneficial for the premature neonate, conferring trophic support and perhaps mimicking a non-injured postnatal microenvironment with a niche that rescues the cells that form the white matter. Moreover, we determined that there is a time window between the insult and the administration of TSC1, where the results are optimized by maximizing the total number of OLs participating in white matter formation and the restoration of CNS function.

### 4.3. Modeling and Rescue of Perinatal White Matter Damage

The neonatal brain is a rapidly changing structure, developing glia and synapses. Because neural connections are still developing at birth and myelination is in its most active period, many medications for treatment and protection in the adult CNS are ineffective and could even be deleterious, as some adult treatments are toxic to neonatal brains [48]. In addition to preventing or delaying premature birth, which is considered to be the most important step in reducing the risk of PVL, no treatments have been approved for use in human PVL patients. Nonetheless, significant strides have been made to develop treatments for the protection of the nervous system. Researchers have examined the potential of synthetic neuroprotection to minimize the extent of these lesions in young patients exposed to ischemic conditions [28]. In this study, the effects of NMDA were less severe in the presence of TSC1, sparing brain tissue and reducing ventriculomegaly through the neuroprotection of pre-existing Olig2-expressing OLPs, CNPase-expressing OLs and medium-sized spiny neurons. Moreover, brains treated with NMDA in the presence of TSC1 did not show the upregulation in the expression of heat shock protein 90 (HSP-90). Like other HSPs, the molecular chaperone HSP-90 is involved in the folding, stabilization and binding of many proteins and is essential for maintaining the integrity of many signaling cascade pathways in response to cellular stress through aberrant expression and/or mutation [49,50]. HSP-90 was first isolated after extracting proteins from cells under various stresses, including heat, dehydration or other stressors that induce protein denaturation [51]. Moreover, HSP-90 also has essential functions in unstressed cells. Pratt and colleagues reported that monoclonal antibodies raised against glucocorticoid receptor (GR) identified the receptor and a 90 kD protein that cross-reacted with antibodies raised against HSP-90 [52], thus identifying HSP-90 as a steroid hormone receptor chaperone. Steroid hormone receptors are a class of transcription factors activated through binding to steroid agonists and were some of the first proteins actively studied as HSP-90 clients. Since then, HSP-90 has been shown to bind strongly to the apo-form of many steroid hormone receptors, including estrogen and androgen receptors [53]. It is now understood that HSP-90-dependent receptors require HSP-90 to bind to steroids [54]. Moreover, HSP-90 mediates steroid receptor maturation, and ATP hydrolysis through HSP-90 is required for efficient protein maturation [55]. Steroid binding controls the activity of all steroid receptors, and for HSP-90-dependent receptors, this step requires HSP-90 activity. When HSP-90 is inhibited, heat shock factor-1 (HSF-1) is activated, leading to the subsequent activation of protective stress-induced HSPs, such as HSP-70 [56,57]. The pharmacological induction of HSF1 through HSP-90 inhibitors in cell culture and animal models is protective against the toxicity induced through pathogenic proteins, ameliorating abnormal transgenic phenotypes in animal models and suppressing protein aggregate formation in several models of neurodegeneration [56,57]. Thus, HSP-90 inhibition is therapeutically effective by specifically degrading misfolded or mutated client proteins that contribute to disease pathology. HSP-90 inhibitors, such as 17-AAG and radicicol, have similar neuroprotective effects as those of HSF-1 and HSP-70 up-regulation [58,59]. The blood-brain barrier permeability presents a limitation [60,61]. 17-AAG is currently in phase II trials as an antitumor compound [62,63], showing neuroprotective effects in preclinical models of Huntington's disease and spinocerebellar ataxias [57,59]. Here, NMDA induced the upregulation of HSP-90 in the white matter, but in the presence of TSC1, NMDA did not induce HSP-90 expression, suggesting that TSC1 plays a role not only on OLP survival and migration but also in regulating HSP-90 expression in white matter regions. Our data from the electrophysiology experiments showed that striatal MSNs were also protected by TSC1, indicating that several cell types benefit from the neuroprotective effects of this compound. In Huntington’s disease, MSNs are particularly vulnerable to the mutation, and the mechanism of cell death involves excitotoxic mechanisms [64]. If NMDA receptors are more sensitive in this disease, then the local administration of TSC1 could serve as a neuroprotective strategy.

The purpose of this study was to examine the potential therapeutic effects of TSC1 after the deleterious effects of glutamate excitotoxicity, and therefore, we did not address the potential mechanisms of action of this cocktail. Studies are currently underway to elucidate the mechanisms underlying the actions of TSC1.

## 5. Conclusions

White matter loss following an excitotoxic insult is not totally irreversible in premature neonates, and the attenuation of the early events with a single intervention with TSC1 administered simultaneously or at 3 days after the NMDA injection reduced brain tissue loss (1) by protecting pre-existing committed Olig2 and CNPase-positive OLs; (2) by enhancing cell proliferation and progression towards mature, functional OLs; and (3) through TSC1. Moreover, striatal MSNs were also protected. Therefore, TSC1 is the first promising intervention to alleviate perinatal white matter loss and to prevent long-term disabilities, such as cerebral palsy and intellectual disabilities.

### Treatment Challenges and Future Directions

Currently, we propose that a single dose of TSC1 would be necessary and sufficient to mitigate cell loss and the enlargement of the ventricle after excitotoxicity. We also defined a developmental window in which the effects of TSC1 are beneficial and demonstrated that even three days after the insult caused by NMDA there is some benefit conferred to the forming white matter. Nonetheless, the simultaneous injection proved to provide the most benefit in terms of preventing tissue loss and enhancing survival of existing progenitors at the time of the insult with a concomitant reduction of ventricular enlargement. These results indicate that the sooner the intervention the more extensive benefit will be conferred to neural progenitors. Thus, after premature birth, the time at which a single TSC1 dose is administered may determine the lifelong impact on white matter formation and function of the CNS in the premature neonate. At present, the main challenge is to deliver the treatment in a non-invasive manner without decreasing its efficacy. We are currently studying various modes of intervention.

## References

[1] Nave K.A. (2010). Myelination and the trophic support of long axons. Nat. Rev. Neurosci..

[2] Blumenthal I. (2004). Periventricular leucomalacia: A review. Eur. J. Pediatr..

[3] Du Plessis A.J., Volpe J.J. (2002). Perinatal brain injury in the preterm and term newborn. Curr. Opin. Neurol..

[4] Goldberg M.P., Ransom B.R. (2003). New light on white matter. Stroke.

[5] Deng W. (2010). Neurobiology of injury to the developing brain. Nat. Rev. Neurol..

[6] Dammann O., Leviton A. (1997). Does prepregnancy bacterial vaginosis increase a mother’s risk of having a preterm infant with cerebral palsy?. Dev. Med. Child Neurol..

[7] Dammann O., Leviton A. (1997). Maternal intrauterine infection, cytokines, and brain damage in the preterm newborn. Pediatr. Res..

[8] Volpe J.J. (2001). Neurobiology of periventricular leukomalacia in the premature infant. Pediatr. Res..

[9] Brunssen S.H., Harry G.J. (2007). Diffuse white matter injury and neurologic outcomes of infants born very preterm in the 1990s. J. Obstet. Gynecol. Neonatal Nur..

[10] Back S.A., Rivkees S.A. (2004). Emerging concepts in periventricular white matter injury. Semin. Perinatol..

[11] Hagberg H., Peebles D., Mallard C. (2002). Models of white matter injury: Comparison of infectious, hypoxic-ischemic, and excitotoxic insults. Ment. Retard. Dev. Disabil. Res. Rev..

[12] Mesples B., Plaisant F., Fontaine R.H., Gressens P. (2005). Pathophysiology of neonatal brain lesions: Lessons from animal models of excitotoxicity. Acta Paediatr..

[13] Rees S., Inder T. (2005). Fetal and neonatal origins of altered brain development. Early Hum. Dev..

[14] Karadottir R., Cavelier P., Bergersen J.H., Attwell D. (2005). NMDA receptors are expressed in oligodendrocytes and activated in ischaemia. Nature.

[15] Salter M.G., Fern R. (2005). NMDA receptors are expressed in developing oligodendrocyte processes and mediate injury. Nature.

[16] Kumar S., Macklin W.B., Gordon M.N., Espinosa de los Monteros A., Cole R., Scully S.A., de Vellis J. (1990). Transcriptional regulation studies of myelin associated genes in *md* mutant rats. Dev. Neurosci..

[17] Espinosa de los Monteros A., Kumar S., Scully R., Cole R., de Vellis J. (1990). Transferrin gene expression and secretion by rat brain cells *in vitro*. J. Neurosci. Res..

[18] Espinosa de los Monteros A., de Vellis J. (1988). Myelin basic protein and transferrin characterize different subpopulations of oligodendrocytes in rat primary glial cultures. J. Neurosci. Res..

[19] Espinosa de los Monteros A., Zhang M., Gordon M.N., Kumar S., Scully S., de Vellis J. (1990). The myelin-deficient rat mutant: Partial recovery of oligodendrocyte maturation *in vitro*. Dev. Neurosci..

[20] Espinosa de los Monteros A., Kumar S., Zhao P., Huang J.C., Nazarian R., Pan T., Scully S., Chang R., de Vellis J. (1999). Transferrin is an essential factor for myelination. Neurochem. Res..

[21] Espinosa-Jeffrey A., Zhao P.M., Awosika O., Huang A., Chang R., de Vellis J. (2002). Transferrin regulates transcription of the *MBP* gene and its action synergizes with IGF-1 to enhance myelinogenesis in the *md* rat. Dev. Neurosci..

[22] Yamaguchi M., Saito H., Suzuki M., Mori K. (2000). Visualization of neurogenesis in the central nervous system using nestin promoter-GFP transgenic mice. Neuroreport.

[23] Espinosa-Jeffrey A., Zhao P., Awosika W., Wu N., Macias F., Cepeda C., Levine M., de Vellis J. (2006). Activation, proliferation and commitment of endogenous, stem/progenitor cells to the oligodendrocyte lineage by a combination of neurotrophic factors in a rat model of dysmyelination. Dev. Neurosci..

[24] Espinosa de los Monteros A., Zhan M., Gordon G.M., Aymie M., Vellis J. (1992). Transplantation of cultured premyelinating oligodendrocytes into normal and myelin-deficient rat brain. Dev. Neurosci..

[25] Ma J., Matsumoto M., Tanaka K., Takebayashi H., Ikenaka K. (2005). An animal model for late onset chronic demyelination disease caused by failed terminal differentiation of oligodendrocytes. Neuron Glia Biol..

[26] Espinosa-Jeffrey A., Hitoshi S., Zhao P., Awosika O., Agbo C., Olaniyan E., Garcia J., Valera R., Thomassian A., Chang W.R. (2010). Functional central nervous system myelin repair in an adult mouse model of demyelination caused by proteolipid protein overexpression. J. Neurosci. Res..

[27] Mallory F.B. (1942). Pathological Technique: A Practical Manual for Workers in Pathological Histology Including Directions for the Performance of Autopsies and for Microphotography.

[28] Fleiss B., Gressens P. (2012). Tertiary mechanisms of brain damage: A new hope for treatment of cerebral palsy?. Lancet Neurol..

[29] Espinosa de los Monteros A., Baba H., Zhao P.M., Pan T., Chang R., de Vellis J., Ikenaka K. (2001). Remyelination of the adult demyelinated mouse brain by grafted oligodendrocyte progenitors and the effect of B-104 cografts. Neurochem. Res..

[30] Miller S.P., Ferriero D.M. (2009). From selective vulnerability to connectivity: Insights from newborn brain imaging. Trends Neurosci..

[31] Sprinkle T.J. (1989). 2′,3′-Cyclic nucleotide 3′-phosphodiesterase, an oligodendrocyte-Schwann cell and myelin-associated enzyme of the nervous system. Crit. Rev. Neurobiol..

[32] Vogel U.S., Thompson R.J. (1988). Molecular structure, localization, and possible functions of the myelin-associated enzyme 2′,3′-cyclic nucleotide 3′-phosphodiesterase. J. Neurochem..

[33] Giulian D., Moore S. (1980). Identification of 2′,3′-cyclic nucleotide 3′-phosphodiesterase in the vertebrate retina. J. Biol. Chem..

[34] Dreiling C.E., Schilling R.J., Reitz R.C. (1981). 2′,3′-Cyclic nucleotide 3′-phosphohydrolase in rat liver mitochondrial membranes. Biochim. Biophys. Acta.

[35] Weissbarth S., Maker H.S., Raes I., Brannan T.S., Lapin E.P., Lehrer G.M. (1981). The activity of 2′,3′-cyclic nucleotide 3′-phosphodiesterase in rat tissues. J. Neurochem..

[36] Baumann N., Pham-Dinh D. (2001). Biology of oligodendrocyte and myelin in the mammalian central nervous system. Physiol. Rev..

[37] Bartlett W.P., Li X.S., Connor J.R. (1991). Expresion of transferring mRNA in the CNS of normal and jimpy mice. J. Neurochem..

[38] Barlett W.P., Li X.S., Williams M., Benkovic S. (1991). Localization of insulin-like growth factor-1 mRNA in murine central nervous system during postnatal development. Dev. Biol..

[39] Escobar O.P., Bongarzone E.R., Soto E.F., Pasquini J.M. (1994). Single intracerebral injection of apo-transferrin in young rats induces increased myelination. Dev. Neurosci..

[40] Adamo A.M., Paez P.M., Escobar-Cabrera O.R., Wolfson M, Franco P.G., Pasquini J.M., Soto E.F. (2006). Remyelination after cuprizone-induced demyelination in the rat is stimulated by apotransferrin. Exp. Neuol..

[41] Saleh M.C., Espinosa de los Monteros A., de Arriba Zerpa G.A., Fontaine I., Piaud O., Djordjijevic D., Baroukh N., Garcia Otin A.L., Ortiz E., Lewis S. (2003). Myelination and motor coordination are increased in transferrin transgenic mice. J. Neurosci. Res..

[42] Fernandez A.M., Torres-Alemán I. (2012). The many faces of insulin-like peptide signalling in the brain. Nat. Rev. Neurosci..

[43] McMorris F.A., Duboi-Dalcq M. (1988). Insulin-like growth factor I promotes cell proliferation and oligodendroglial commitment in rat glial progenitor cells developing *in vitro*. J. Neurosci. Res..

[44] McMorris F.A., McKinnon R.D. (1996). Regulation of oligodendrocyte development and CNS myelinationby growth factors: Prospects for therapy of demyelinating disease. Brain Pathol..

[45] Werner H., Woloschak M., Adamo M., Shen-Orr Z., Roberts C.T., LeRoith D. (1989). Developmental regulation of the rat insulin-like growth factor I receptor gene. Proc. Natl. Acad. Sci. USA.

[46] Madathil S.K., Evans H.N., Saatman K.E. (2010). Temporal and regional changes in IGF-1/IGF-1R signaling in the mouse brain after traumatic brain injury. J. Neurotrauma.

[47] Zapf J., Froesch E.R. (2011). Insulin-like growth factor I actions on somatic growth. Compr. Physiol..

[48] Rosenberg P.A., Dai W., Gan X.D., Ali S., Fu J., Back S.A, Sanchez R.M., Segal M.M., Follett P.L., Jensen F.E. (2003). Mature myelin basic protein-expressing oligodendrocytes are insensitive to kainate toxicity. J. Neurosci. Res..

[49] Marcu M.G., Schulte T.W., Neckers L. (2000). Novobiocin and related coumarins and depletion of heat shock protein 90-dependent signaling proteins. J. Natl. Cancer Inst..

[50] Xiao X., Zuo X., Davis A.A., McMillan D.R., Curry B.B., Richardson J.A., Benjamin I.J. (1999). HSF1 is required for extra-embryonic development, postnatal growth and protection during inflammatory responses in mice. EMBO J..

[51] Prodromou C., Panaretou B., Chohan S., Siligardi G., O’Brien R., Ladbury J.E., Roe S.M., Piper P.W., Pearl L.H. (2000). The ATPase cycle of Hsp90 drives a molecular ‘clamp’ via transient dimerization of the *N*-terminal domains. EMBO J..

[52] Panaretou B., Siligardi G., Meyer P., Maloney A., Sullivan J.K., Singh S., Millson S.H., Clarke P.A., Naaby-Hansen S., Stein R. (2002). Activation of the ATPase activity of hsp90 by the stress-regulated cochaperone aha1. Mol. Cell.

[53] Spence R.D., Hamby M.E., Umeda E., Itoh N., Du J., Wisdom S., Cao Y., Bondar G., Lam J., Ao Y. (2011). Neuroprotection mediated through estrogen receptor-alpha in astrocytes. Proc. Natl. Acad. Sci. USA.

[54] Passinen S., Valkila J., Manninen T., Syvala H., Ylikomi T. (2001). The *C*-terminal half of Hsp90 is responsible for its cytoplasmic localization. Eur. J. Biochem..

[55] Picard D., Yamamoto K.R. (1987). Two signals mediate hormone-dependent nuclear localization of the glucocorticoid receptor. EMBO J..

[56] Dickey C.A., Eriksen J., Kamal A., Burrows F., Kasibhatla S., Eckman C.B., Hutton M., Petrucelli L. (2005). Development of a high throughput drug screening assay for the detection of changes in tau levels—Proof of concept with HSP90 inhibitors. Curr. Alzheimer Res..

[57] Fujikake N., Nagai H., Popiel H.A., Okamoto Y., Yamaguchi M., Toda T. (2008). Heat shock transcription factor 1-activating compounds suppress polyglutamine-induced neurodegeneration through induction of multiple molecular chaperones. J. Biol. Chem..

[58] Auluck P.K., Meulener M.C., Bonini N.M. (2005). Mechanisms of suppression of α-synuclein neurotoxicity by geldanamycin in drosophilia. J. Biol. Chem..

[59] Waza M., Adachi H., Katsuno M., Minamiyama M., Tanaka F, Sobue G. (2006). Alleviating neurodegeneration by an anticancer agent: An Hsp90 inhibitor (17-AAG). Ann. N. Y. Acad. Sci..

[60] Chiosis G., Tao H. (2006). Purine-scaffold Hsp90 inhibitors. IDrugs.

[61] Taldone T., Gozman A., Maharaj R., Chiosis G. (2008). Targeting Hsp90: Small-molecule inhibitors and their clinical development. Curr. Opin. Pharmacol..

[62] Heath E.L., Gaskins M., Pitot H.C., Pili R., Tan W., Marschke R., Liu G., Hillman D., Sarkar F., Sheng S. (2005). A phase II trial of 17-allylamino-17-demethoxygeldanamycin in patients with hormone-refractory metastatic prostate cancer. Clin. Prostate Cancer.

[63] Ramanathan R.K., Trump D.L., Eiseman J.L., Belani C.P., Agarwala S.S., Zuhowski E.G., Lan J., Potter D.M., Ivy S.P., Ramalingam S. (2005). Phase I pharmacokinetic-pharmacodynamic study of 17-(allylamino)-17-demethoxygeldanamycin (17AAG, NSC 330507), a novel inhibitor of heat shock protein 90, in patients with refractory advanced cancers. Clin. Cancer Res..

[64] DiFiglia M. (1990). Excitotoxic injury of the neostriatum: A model for Huntington’s disease. Trends Neurosci..

